# Structure and composition of arbuscular mycorrhizal fungal community associated with mango

**DOI:** 10.3389/fpls.2025.1578936

**Published:** 2025-05-08

**Authors:** Cuifeng Yang, Zheng Teng, Zhibo Jin, Qiufei Ouyang, Lingling Lv, Xianbin Hou, Muzammil Hussain, Zhengjie Zhu

**Affiliations:** ^1^ College of Agriculture and Food Engineering, Baise University, Baise, China; ^2^ Guangxi Key Laboratory of Biology for Mango, Baise, China; ^3^ College of Subtropical Characteristic Agricultural Industry, Baise, China

**Keywords:** AMF community, mango, microbial diversity, planting years, seasonal dynamic, soil properties

## Abstract

Mango (*Mangifera indica* L.) is an important fruit crop with significant economic value in tropical and subtropical areas globally. Arbuscular mycorrhizal fungal (AMF) symbiosis is vital for mango trees growth, and the detailed understanding of various (a)biotic factors that influence AMF community composition is crucial for sustainable crop production. To date, there is little information available on how do different seasons and plant age influence the AMF community composition associated with mango. Using high-throughput amplicon sequencing, we examined AMF community diversity and composition in the rhizosphere of mango from two distinct orchards during spring (C_BY and C_YL) and autumn (Q_BY and Q_YL), which differed in age (10 and 28 years). The results revealed a notable variation in the number of observed species between two 28-years-old mango orchards (C_BY28 vs C_YL28 and Q_BY28 vs Q_YL28) during both the spring and autumn seasons. However, the comparison of 10-years-old and 28-years-old mangoes showed no significant shift in the diversity and richness of AMF. At the taxonomic level, *Glomus* was the absolute dominant genus in AMF community. The correlation analysis between species abundance and soil nutrients showed that the level of phosphorus, potassium and their available forms (AP, AK) significantly affect AMF community. Furthermore, the P, AP, and AK contents were found positively correlated with the dominant AMF molecular virtual species *Sclerocystis sinuosa*. These findings indicate the response characteristics of mango rhizosphere AMF community to soil nutrients, providing scientific basis for precise regulation of soil environment to improve mango tree growth and production.

## 1 Introduction

The agricultural economy on a global scale heavily depends on tropical fruits, and their development is influenced by several factors, notably the interplay between soil nutrients and native microbial communities. Arbuscular mycorrhizal fungi (AMF) are beneficial group of microorganisms commonly found in the soils of diverse ecosystems, capable of establishing symbiotic association with the root systems of many tropical fruit species. AMF can effectively promote the absorption and utilization of mineral nutrients by plants through the formation of a complex mycelial networks (Hartoyo and Trisilawati, 2021), which helps with the growth and development of tropical fruits, enhances plant resistance to biotic and abiotic stresses (Mickan et al., 2016; Boldt-Burisch et al., 2018), affects the secondary metabolism process of tropical fruits, and directly or indirectly affects the community structure, diversity, and productivity of different ecosystems (Meddad-Hamza et al., 2010; Zhang et al., 2015). AMF also provides a low-cost solution for stress resistant cultivation of tropical fruits under climate change and is currently a hot topic in exploring and utilizing beneficial microbial resources in tropical fruits. Studies have shown that AMF community composition is mainly influenced by factors such as management measures, host plants, and environmental conditions (Jia et al., 2023).

Mango (*Mangifera indica* L.) is an important fruit trees widely grown in tropical and subtropical regions (Xie et al., 2025). China is the world’s second largest mango producing country (Guo et al., 2021), and Baise City, situated in the Guangxi Zhuang Autonomous Region of China, is a major hub for mango production with a total harvest of 1.22 million tons (Guo et al., 2021). The Baise mango is recognized as a national geographical indication agricultural product. However, the increase in human activities and non-scientific agricultural practices over an extended period has greatly affected the soil biodiversity in mango growing regions. For example, according to one regional study, the excessive use of fertilizers and traditional farming approaches have led to considerable harm to the soil ecosystem in mango production areas of Baise city (Ou et al., 2021). Generally, soil nutrients like nitrogen (N), phosphorus (P), potassium(K) and calcium (Ca) significantly alter the diversity and composition of AMF communities (de Pontes et al., 2017). Changes in soil pH may also directly and indirectly shift AMF diversity (Svenningsen et al., 2018). Therefore, the ongoing challenges to soil fertility threaten AMF community and sustainable mango production, significantly impeding the developmental progress of the mango industry (Govindan et al., 2020; Mohammed et al., 2022).

AMF communities associated with plants have direct and profound impact on the nutrient absorption efficiency, stress resistance, and ultimately fruit yield and quality of mangoes (Singh et al., 2024). Previous studies have identified a number of AMF taxa such as *Acaulospora*, *Diversispora*, *Glomus*, *Gigaspora*, *Paraglomus* and *Rhizophagus* from mango orchards using both traditional and high-throughput sequencing methods (Teixeira-Rios et al., 2018; Li et al., 2022). However, the questions regarding how the plants age and seasonal dynamics affect the diversity and composition of mango AMF community remains unanswered. Herein, we hypothesized that the age of mango trees and seasonal variation may significantly affect the structure and composition of AMF community. To test this hypothesis, we obtained rhizosphere samples from 10 and 28 years old mango plants across the spring and autumn seasons. Our specific objectives were to (a) assess the diversity and richness of AMF populations in two separate mango orchards in Baise city (b) decipher the structure and composition of the AMF community in the mango rhizosphere throughout different seasons and years of planting, and (c) understand how the structure of AMF community relates to the nutrient composition of the soil. This study offers valuable insights into the composition of AMF communities and their connection to soil health in mango orchards located in Baise city of the Guangxi Zhuang Autonomous Region of China.

## 2 Materials and methods

### 2.1 Sample collection

In November 2022 (autumn) and May 2023 (spring), the root samples with attached rhizosphere soil were collected from two mango orchards differed in age (10 and 28 years) located in Baise City, Guangxi Zhuang Autonomous Region, China. One orchards is situated in Baiyu Town (BY), Tianyang District (106°22′14″–107°08′32″ E, 23°28′20″–24°06′55″ N) characterized by an average annual temperature ranging from 18 to 22 °C, yearly rainfall between 1,100 and 1,250 mm, a frost-free duration of 307 to 352 days, and a total annual sunshine of 1,906.6 hours. The second orchard is located in Yongle Town (YL), Youjiang District (106°07′–106°56′ E, 23°33′–24°18′ N) characterized by an average annual temperature of 22 °C, total annual precipitation of 1,350 mm, a frost-free period of 360 days, and an annual sunshine duration of 1,633 hours. There is a significant heterogeneity in the geographical and climatic conditions between the two sampling sites, which is conducive to analyzing the construction of AMF communities and the temporal and spatial patterns of their responses to environmental factors. Five independent replicate samples were collected from 10-and 28-year-old mango plantations respectively (**Supplementary Table 1**), reducing the interference of microhabitat heterogeneity and meet the sample size requirements for subsequent statistical tests. The sample names in this study are as follow: C_BY10, 10yr old orchard in Baiyu Town during spring; C_BY28, 28yr old orchard in Baiyu Town during spring; C_YL10, 10yr old orchard in Yongle Town during spring; C_YL28, 28yr old orchard in Yongle Town during spring; Q_BY10, 10yr old orchard in Baiyu Town during autumn; Q_BY28, 28yr old orchard in Baiyu Town during autumn; Q_YL10, 10yr old orchard in Yongle Town during autumn; Q_YL28, 28yr old orchard in Yongle Town during autumn. In order to collect root samples through the five point sampling method, we first removed large gravel, dead branches, and leaves from the surface to ensure that the collected samples can truly reflect the rhizosphere soil conditions and avoid interference from debris. Next, excavate the roots to a depth of 5-30 cm, and then gently knock off the root with attached rhizosphere soil that is tightly bound to the root system and place it in a sterile bag. After the soil was bought back to the laboratory, the rhizosphere soil was collected in accordance with the methods described by Hussain et al. (2024). It was then divided into two parts, with one part used for DNA extraction and subsequent AMF molecular identification, and the other part for the analysis of soil chemical properties.

### 2.2 Determination of soil chemical properties

The soil chemical properties were determined according to soil agrochemical analysis (Bao, 2000). Soil pH was determined by electrode method. The contents of total nitrogen (N), total phosphorus (P) and total potassium (K) in soil were determined by Kjeldahl method, perchloric acid sulfuric acid method and alkali fusion atomic absorption spectrometry, respectively. The contents of alkali hydrolyzed nitrogen (HN), available phosphorus (AP) and available potassium (AK) were determined by alkali hydrolysis diffusion method, sodium bicarbonate extraction - molybdenum antimony anti colorimetry method and ammonium acetate extraction - Flame photometric method, respectively. Organic matter (OM) content was determined by potassium dichromate external heating method.

### 2.3 DNA extraction, PCR amplification and Illumina sequencing

The DNA from rhizosphere soil was extracted using the cetyltrimethylammonium bromide (CTAB) method to examine AMF communities as described previously (Yurkov et al., 2023), due to its cationic detergent properties that effectively bind and precipitate polysaccharide-protein complexes, particularly suitable for complex soil environments containing humic acids and inhibitors. After adjusting the DNA concentration to 20 ng/µL, the AMF 18S rDNA region was amplified using the specific barcoded primers AMV4.5NF (5’-AAGCTCGTAGTTGAATTTCG-3’) and AMDGR(5’- CCCAACTATCCCTATTAATCAT-3’) in a Veriti thermal cycler (Applied Biosystems). The PCR reaction mixture (20 µL) includes: 5 × fastpfu buffer 4.0 µL, 2.5 mmol/L dNTPs 2.0 µL, 5 µmol/L upstream and downstream primers 0.8 µL, fastpfu polymerase 0.4 µL, BSA 0.2 µL, template DNA 0.01 µL, ddH_2_O make up to 20.0 µL. Amplification procedure: pre-denaturation at 95 °C for 3 min; 35 cycles were performed at 95 °C for 30 s, 55 °C for 30 s, and 72 °C for 45 s; extend at 72 °C for 10 min. The amplified product from PCR was purified with the PCR Clean-Up Kit (YuHua, Shanghai, China), and the final concentration of the DNA was determined by Qubit 4.0 (Thermo Fisher Scientific, USA). Finally, the paired-end sequencing was performed using the Illumina Nextseq 2000 platform by Majorbio Bio-Pharm Technology Co. Ltd. (Shanghai, China). The raw sequencing reads were deposited into the NCBI Sequence Read Archive (SRA) database (Accession Number: PRJNA1224724).

### 2.4 Bioinformatics analyses of amplicon sequencing

Quality control on double-ended raw sequencing data is carried out using the fastp software tool (https://github.com/OpenGene/fastp, Version 0.19.6). The sequencing reads were merged using FLASH (http://www.cbcb.umd.edu/software/flash, Version 1.2.11) and the resulting high-quality sequences were then de-noised using DADA2. The classification of amplicon sequence variants (ASVs) was carried out utilizing the Naive Bayes consensus taxonomy classifier that is integrated into QIIME2. The calculations for rarefaction curves and alpha diversity metrics, such as the Sobs and Shannon indices, were performed using Mothur version 1.30.1. The Wilcoxon rank sum test was employed to assess the differences in alpha diversity among the groups. To determine the relative significance of certainty and randomness in the AMF community assembly, we applied a zero model (999 randomizations) for calculating the β-nearest taxonomic unit index (β NTI) through the R package ‘icamp’. The analysis of microbial typing conducted based on the proportion of the microbial community at a chosen classification tier, utilizing the Jensen Shannon Distance (JSD) for equidistant calculations and applying PAM (Partitioning Around Medoids) for clustering. The Calinski Harabasz index is employed to determine the ideal number of clusters (K), followed by principal coordinates analysis (PCoA, K ≥ 2) for visualization purposes. This analysis is performed using R version 3.3.1 with the packages ‘ade4’, ‘cluster’, and ‘clusterSim’. Venn diagram was created using the R package VennDiagrams. Circos diagrams was created using Circos-0.67-7 (http://circos.ca/). Species differential analysis was performed using R package ‘stat’ and the Wilxocon rank sum test was used to identify the differences in mean values of different groups. We then conducted a distance-based redundancy analysis (db-RDA) to assess the influence of soil chemical properties on the community structure of AMF in the soil. A numerical matrix was created by evaluating the correlation coefficient of environmental factors in relation to selected species and presented visually as a heatmap. The functional prediction of 18S rDNA sequencing reads was performed using PICRUSt2.

## 3 Results

### 3.1 Analysis of chemical properties of rhizosphere soil in mango orchard

The chemical properties of rhizosphere soil in mango orchards with different seasons, planting locations, and planting years exhibited strong heterogeneity (**Supplementary Tables 2**, **3**). The differences in various indicators between spring (C_BY10, C_BY28, C_YL10 and C_YL28) and autumn (Q_BY10, Q_BY28, Q_YL10 and Q_YL28) did not show a clear consistent pattern, with only the OM content in autumn being significantly lower than that in spring. For the same production area, the soil fertility varies with different planting years. The N and OM content in the 28 year old plantation (C_BY28) were significantly higher than those in the 10-year plantation (C_BY10). In Yongle Town, the K content in the 28 year old plantation (Q_YL28) was more than that in the 10-year plantation (Q_YL10). With an increasing planting year, the content of N, OM, K and other nutrients in the soil increased. There were differences in soil fertility indicators between mango orchards in Baiyu Town and Yongle Town. The soil pH of sample C_BY28 was higher than that of C_YL28. The N content of C_YL10 was more than that of C_BY10, and the N content of Q_YL28 was high than that of Q_BY28. The P content of Q_BY10 was more than that of Q_YL10. However, the K content in Yongle Town was significantly higher than that in Baiyu Town. In addition, the AP content in Baiyu Town in autumn was more than that in Yongle Town. After 10 years of planting, the AK content in Baiyu Town was high than that in Yongle Town. This indicates that the chemical properties of the rhizosphere soil in mango orchards vary depending on the planting location, which may be closely related to agronomic measures such as fertilization.

### 3.2 Alpha diversity, community structure and microbial typing analysis

**Figure 1:**
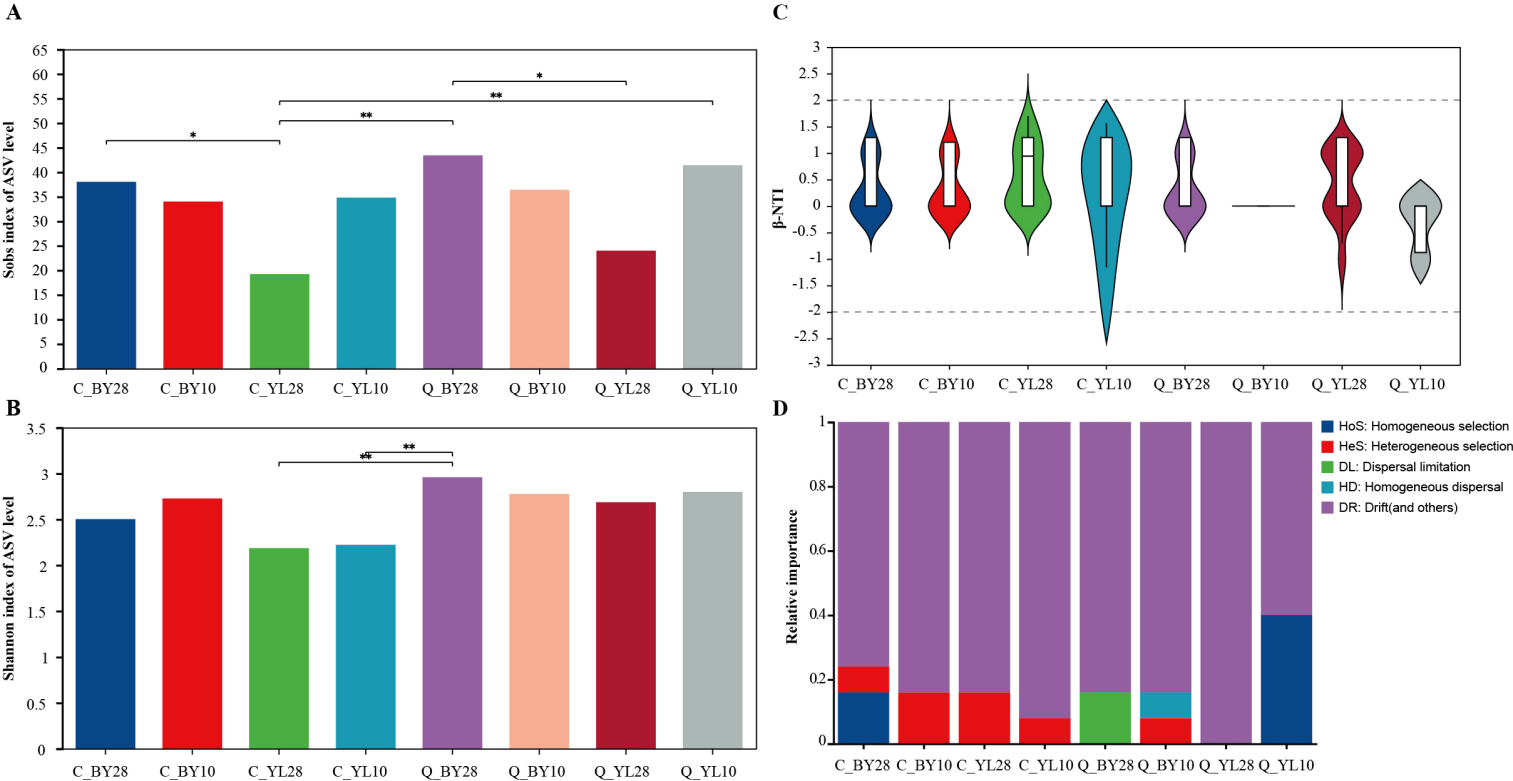
Diversity and community structure of AMF community in mango rhizosphere soil. **(A)** Sobs index of AMF in rhizosphere soil of mango orchard, **(B)** Shannon index of AMF in rhizosphere soil of mango orchard, and **(C)** Analysis of β-NTI community structure of AMF in rhizosphere soil of mango orchard. **(D)** Relative importance of different ecological processes of mango rhizosphere AMF community. Note: 0.01<P ≤ 0.05 is marked as *, 0.001<P ≤ 0.01 is marked as * *, the same applies below. C_BY10, 10yr old orchard in Baiyu Town during spring; C_BY28, 28yr old orchard in Baiyu Town during spring; C_YL10, 10yr old orchard in Yongle Town during spring; C_YL28, 28yr old orchard in Yongle Town during spring; Q_BY10, 10yr old orchard in Baiyu Town during autumn; Q_BY28, 28yr old orchard in Baiyu Town during autumn; Q_YL10, 10yr old orchard in Yongle Town during autumn; Q_YL28, 28yr old orchard in Yongle Town during autumn.

The total reads retrieved from the sequencing data were enough to reflect the vast majority of microbial diversity information in the sample (**Supplementary Figure 1**). In addition, the Coverage Index of each sample was close to 1, indicating that the sequencing results covered all microbial groups in the sample, providing enough data for downstream analysis (**Supplementary Figure 2**). The findings from the Rank Abundance curve indicated that the different seasons (spring and autumn), planting years (10 years and 28 years), and locations (BY and YL) may have important impacts on the relative abundance distribution of microorganisms at the genus level (**Supplementary Figure 3**). The Sobs index values in C_YL28 was lowest as compared to C_BY28, indicating a relative significant different between two 28-years-old mango orchards (**Figure 1A**). The Sobs index values for sample Q_BY28 was also highest than that of Q_YL28, suggesting that despite the seasonal variations, these orchards hosted distinct groups of microbial species. Furthermore, the results of the Shannon index (**Figure 1B**) showed that the sample Q_BY28 had highest AMF diversity than that of C_YL28 and C_YL10, indicating that AMF diversity is relatively low and the evenness of species distribution is not uniform. The results of β-NTI analysis (β-Nearest Taxon Index analysis) showed that the range of β-NTI values for each sample was -1.22~1.71, within the range of |β-NTI |<2, indicating that stochastic processes dominate the assembly of AMF communities in the rhizosphere soil of mango orchards, and they may have different response modes when facing environmental changes (**Figures 1C, D**).

**Figure 2:**
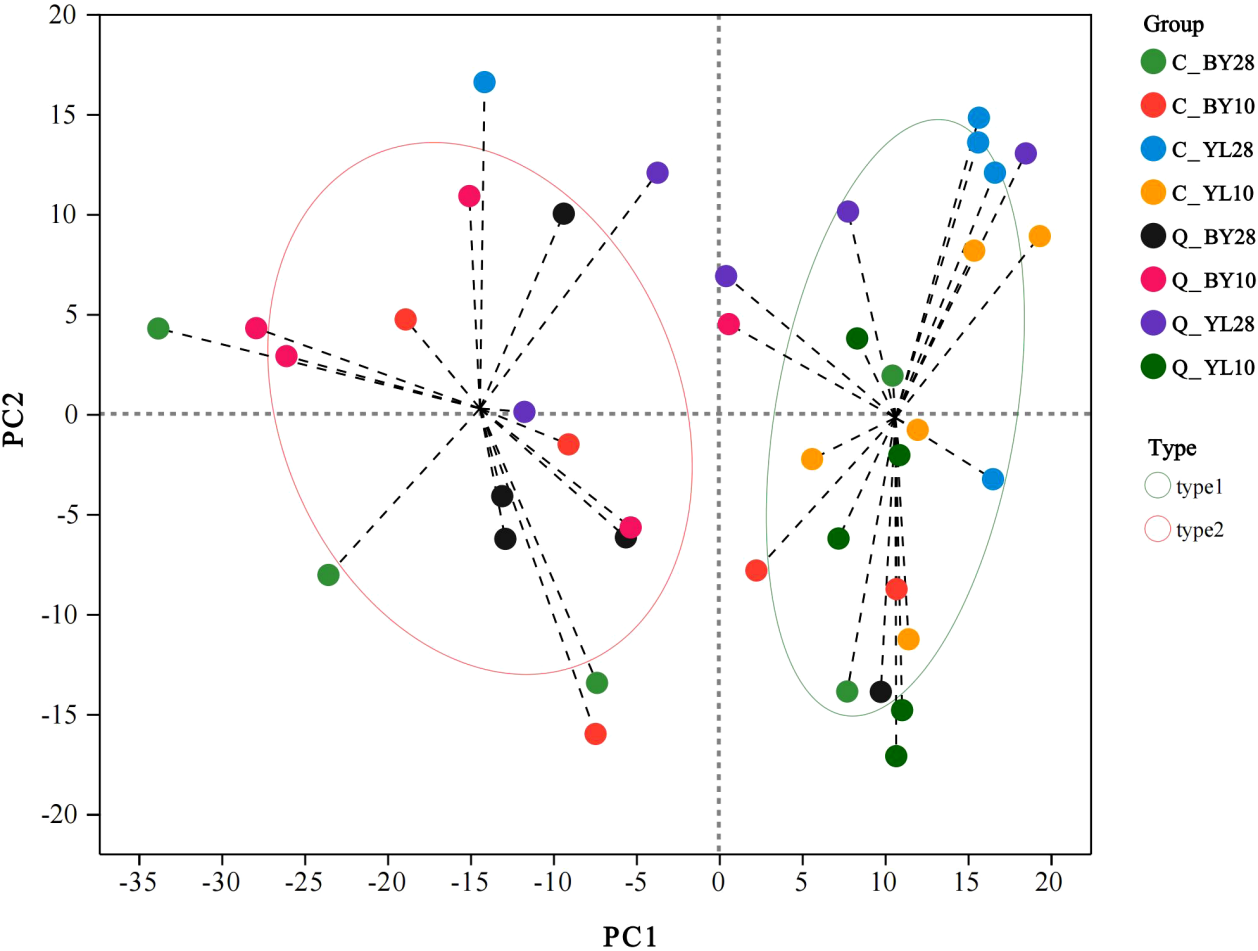
Microbial community typing analysis of AMF in rhizosphere soil of mango orchard. C_BY10, 10yr old orchard in Baiyu Town during spring; C_BY28, 28yr old orchard in Baiyu Town during spring; C_YL10, 10yr old orchard in Yongle Town during spring; C_YL28, 28yr old orchard in Yongle Town during spring; Q_BY10, 10yr old orchard in Baiyu Town during autumn; Q_BY28, 28yr old orchard in Baiyu Town during autumn; Q_YL10, 10yr old orchard in Yongle Town during autumn; Q_YL28, 28yr old orchard in Yongle Town during autumn.

The results of microbial typing analysis at the genus level showed that the AMF community in the rhizosphere of mango orchards was mainly divided into two dominant types (type 1, type 2). Within the confidence interval, the samples of type 1 were concentrated at the sampling point of YL origin, and the samples of type 2 were concentrated at the sampling point of BY origin, indicated that the sampling points of YL origin and BY origin each have one dominant microbial community (**Figure 2**). Among them, the sample size in type 1 was larger than that in type 2, suggesting that type 1 may contain some microbial genera with strong competitiveness, which may be in a relatively advantageous position in the community and play important roles in ecological processes such as nutrient transformation and organic matter decomposition. Type 2 may represent another microbial genus with different ecological strategies, with relatively low abundance in the community, but may still have irreplaceable functions in certain special ecological niches or specific ecological processes.

### 3.3 AMF community composition analysis in rhizosphere soil of mango orchard

**Figure 3:**
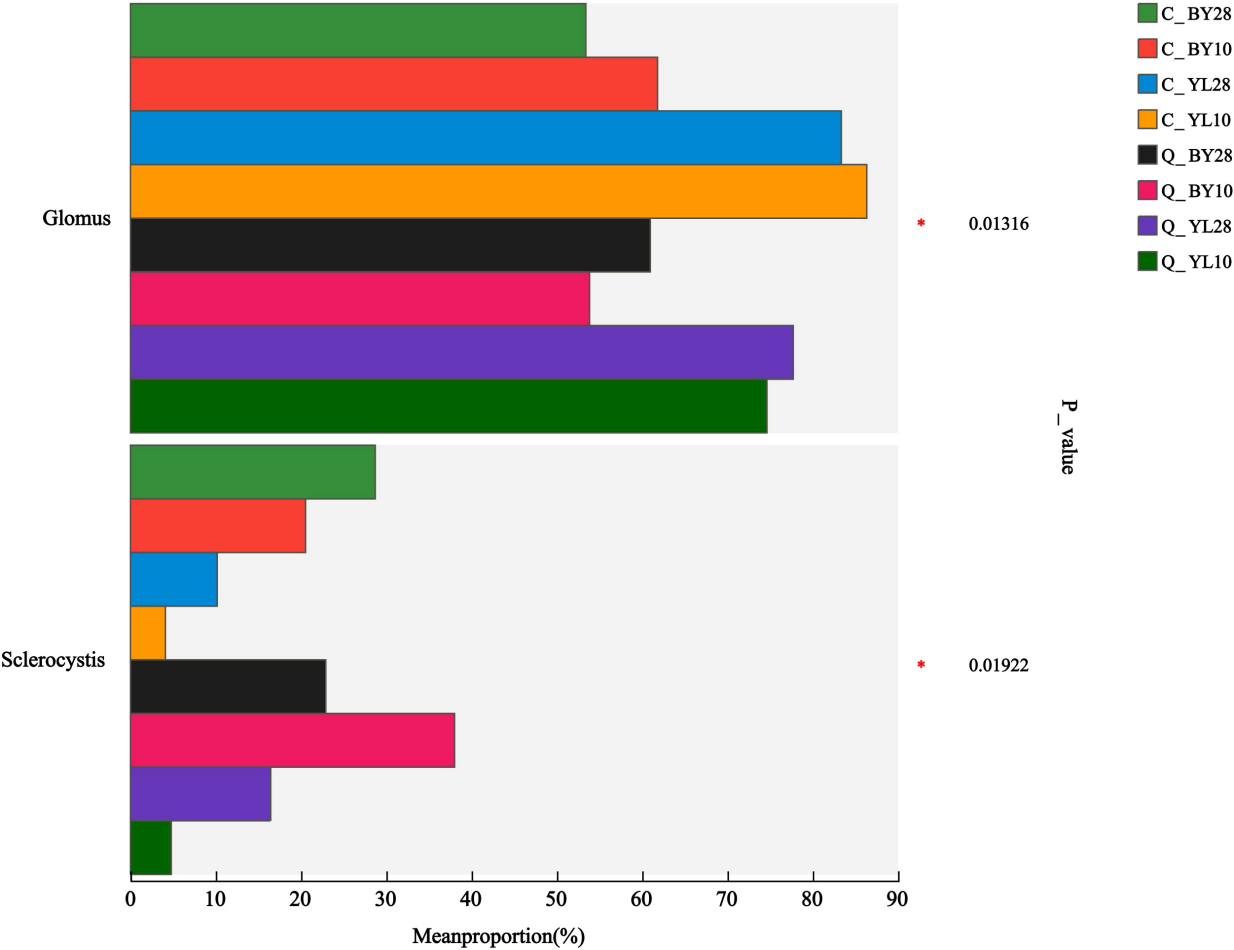
Differences in relative abundance of AMF in rhizosphere soil of mango orchard (genus level). C_BY10, 10yr old orchard in Baiyu Town during spring; C_BY28, 28yr old orchard in Baiyu Town during spring; C_YL10, 10yr old orchard in Yongle Town during spring; C_YL28, 28yr old orchard in Yongle Town during spring; Q_BY10, 10yr old orchard in Baiyu Town during autumn; Q_BY28, 28yr old orchard in Baiyu Town during autumn; Q_YL10, 10yr old orchard in Yongle Town during autumn; Q_YL28, 28yr old orchard in Yongle Town during autumn.

AMF community in the rhizosphere soil of mango orchards shared three genera across samples, namely *Glomus*, *Sclerocystis*, and unclassified_f_*Glomeraceae* (**Supplementary Figure 4A**). There were no unique genera observed in each sample. While at the species level, there were a total of 5 dominant AMF molecular virtual species shared between samples, including sp.*VTX00387*, sp.*VTX00400*, unclassified_g_*Glomus*, *S. sinuosa*, and unclassified_f_*Glomeraceae* (**Supplementary Figure 4B**). The construction of the Circos plot at the genus level showed that the *Glomus* genus dominates the AMF community (**Supplementary Figure 5A**). The average relative abundance of *Glomus* and *Sclerocystis* was significantly different among the groups, with p-values of 0.01316 and 0.01922, respectively (**Figure 3**).

**Figure 4:**
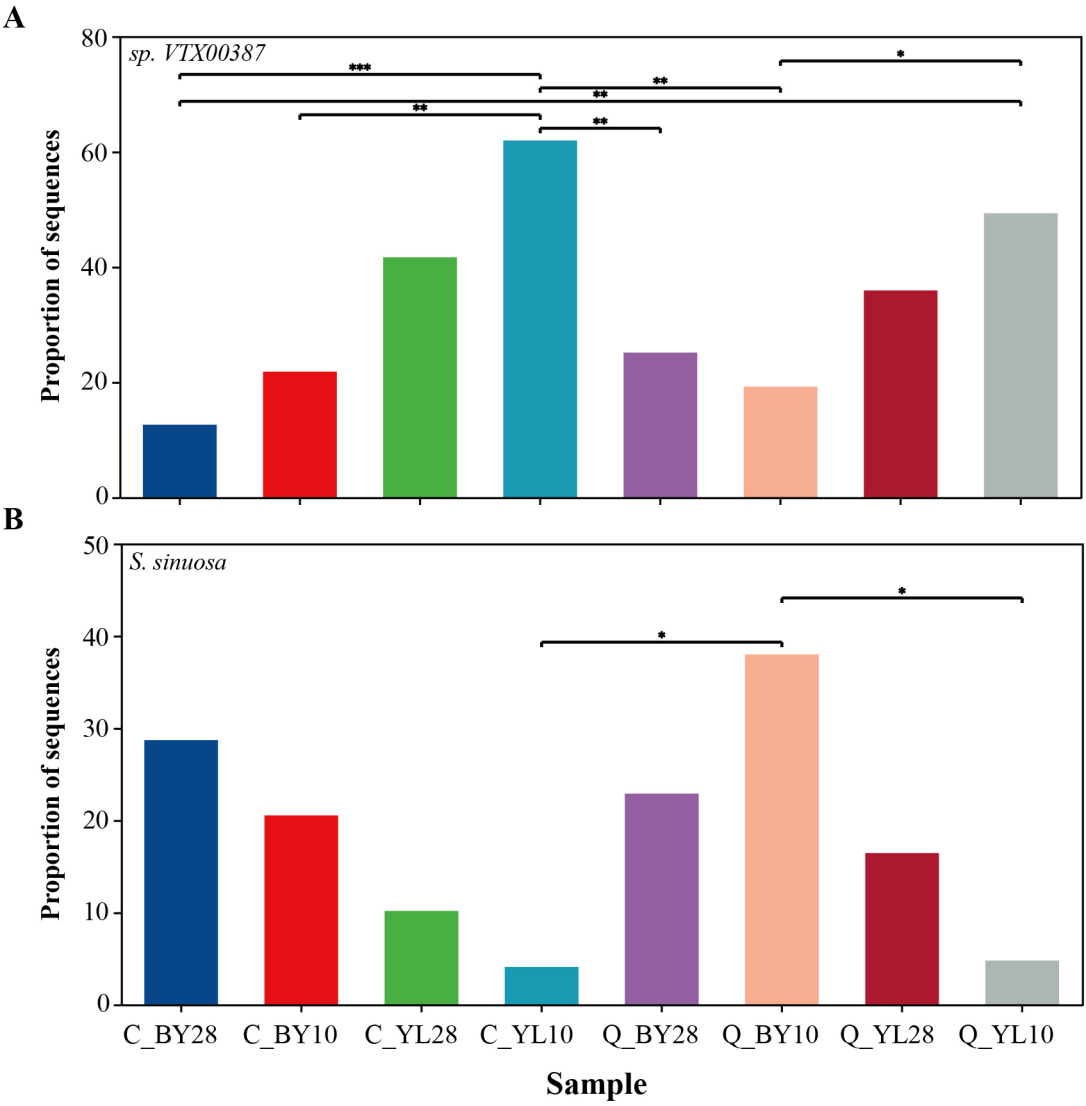
Proportion of sequences of two dominant AMF genera associated with mango rhizosphere soil. **(A)** The proportion of sequences of sp. VTX00387, and **(B)** S. sinuosa in mango rhizosphere soil. Note: 0.01<P ≤ 0.05 is marked as *0.001<P ≤ 0.01 is marked as **P ≤ 0.001 is marked as *** the same applies below. C_BY10, 10yr old orchard in Baiyu Town during spring; C_BY28, 28yr old orchard in Baiyu Town during spring; C_YL10, 10yr old orchard in Yongle Town during spring; C_YL28, 28yr old orchard in Yongle Town during spring; Q_BY10, 10yr old orchard in Baiyu Town during autumn; Q_BY28, 28yr old orchard in Baiyu Town during autumn; Q_YL10, 10yr old orchard in Yongle Town during autumn; Q_YL28, 28yr old orchard in Yongle Town during autumn.

At the species level (**Supplementary Figure 5B**), the molecular virtual species sp.*VTX00387* was the main dominant species in sample C_YL10, with a sequence proportion of up to 61.94%. Further statistical analysis showed that the difference between sample C_YL10 and sample C_BY28 has reached an extremely significant level, which means that there was a fundamental difference in the existence of sp.*VTX00387* between these two samples. At the same time, there were significant differences between sample C_YL10 and samples C_BY10, Q_BY28, and Q_BY10; and between Q_YL10 and C_BY28, reflecting variation in the abundance or activity of the strain in these different sample environments (**Figure 4A**).

For *S. sinuosa*, sample Q_BY10 was a dominant sample, with a sequence proportion of 38.00% and there was a significant difference between sample Q_BY10 and samples C_YL10 and Q_YL10, indicating that the distribution of this species in different samples was not random, but was constrained by specific environmental conditions, resulting in significant differences in its proportion in different samples (**Figure 4B**). Further analysis revealed a negative correlation between the sequence proportions of sp.*VTX00387* and *S. sinuosa* in each sample. In the AMF microbial community of mango orchard rhizosphere soil, the proportions of sp.*VTX00387* and *S. sinuosa* were in a “competitive” state. When the proportion of sp.*VTX00387* decreased, the proportion of *S. sinuosa* increased (**Figures 4A, B**).

### 3.4 Correlation analysis of AMF in rhizosphere soil of mango orchard

**Figure 5:**
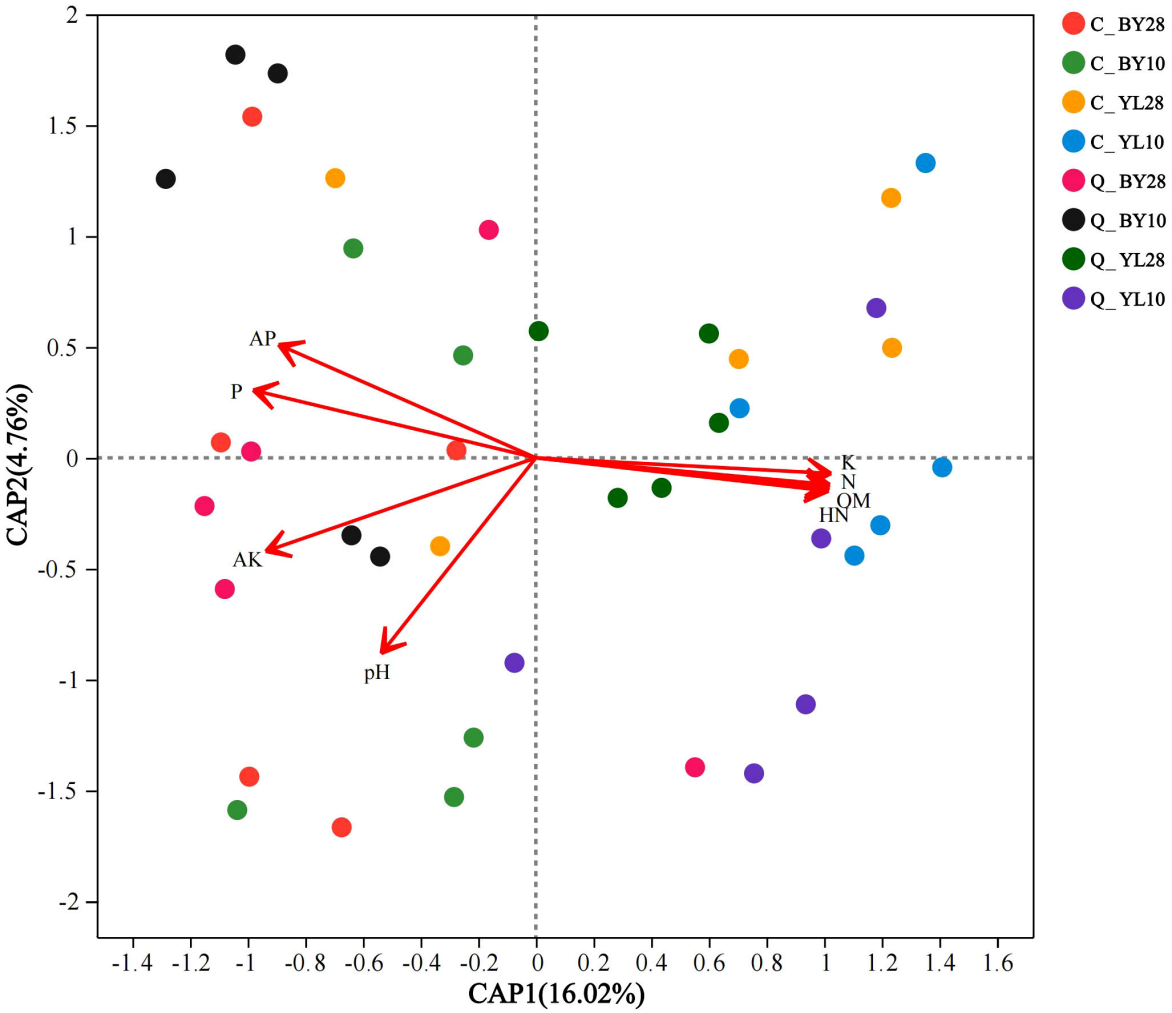
db-RDA analysis of AMF and soil chemical properties in the rhizosphere of mango orchard. C_BY10, 10yr old orchard in Baiyu Town during spring; C_BY28, 28yr old orchard in Baiyu Town during spring; C_YL10, 10yr old orchard in Yongle Town during spring; C_YL28, 28yr old orchard in Yongle Town during spring; Q_BY10, 10yr old orchard in Baiyu Town during autumn; Q_BY28, 28yr old orchard in Baiyu Town during autumn; Q_YL10, 10yr old orchard in Yongle Town during autumn; Q_YL28, 28yr old orchard in Yongle Town during autumn.

At the species level, the db-RDA (distance-based redundancy analysis) analysis of AMF and soil chemical properties in the rhizosphere soil of mango orchards revealed that the two axes of RDA explained 20.78% of the variation in the AMF community, indicating a certain degree of correlation between soil chemical properties and the AMF community (**Figure 5**).

**Figure 6:**
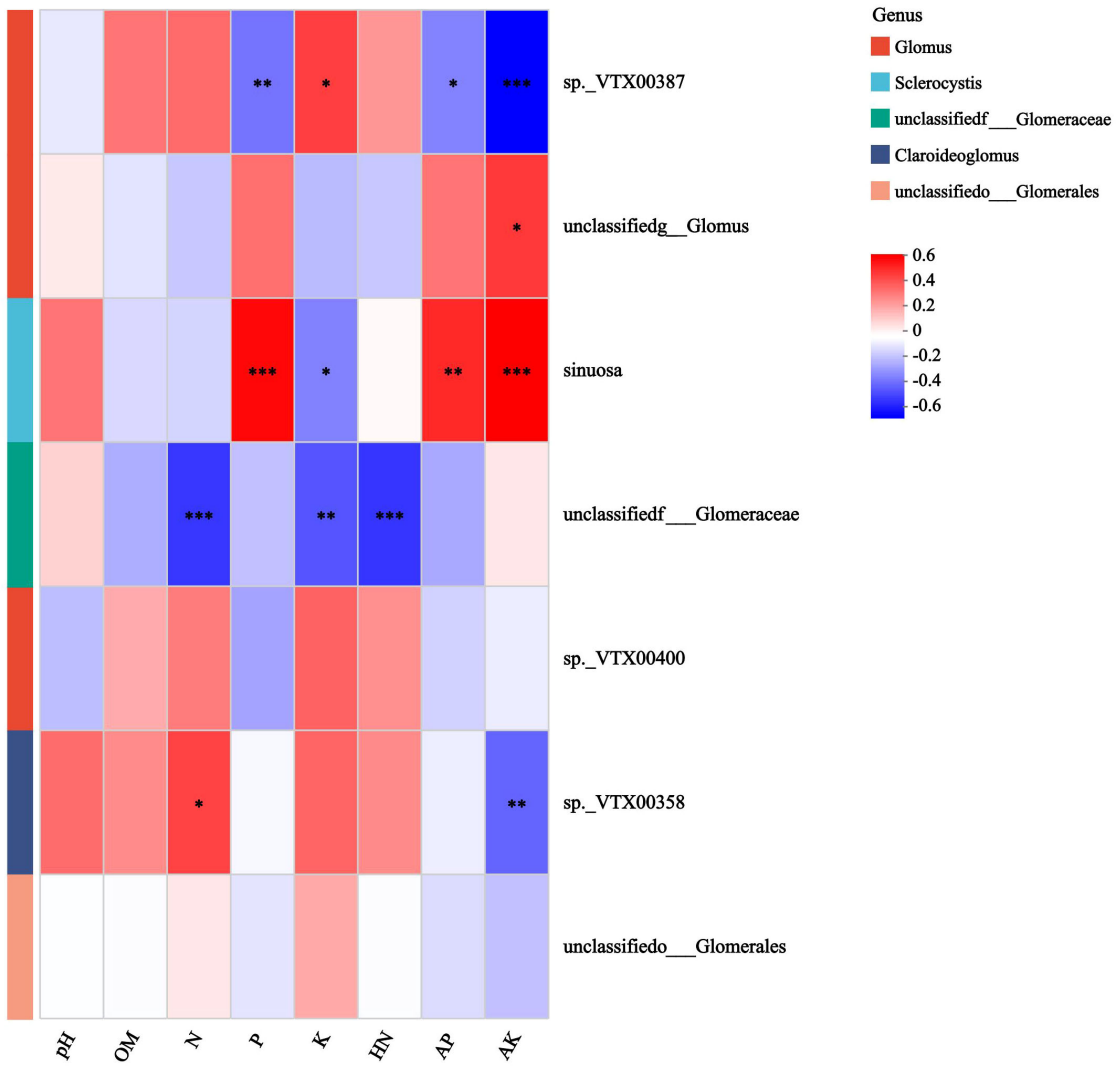
Spearman correlation heatmap of AMF and soil chemical properties in the rhizosphere of mango orchard. * 0.01 < P ≤ 0.05, ** 0.001 < P ≤ 0.01, *** P ≤ 0.001.

Further analysis reveals that the canonical Axis 1 (CAP1) coefficient of K was 0.9976, indicating that K had a strong explanatory power for changes in microbial community structure, explaining nearly 54.18% of the induced changes (**Supplementary Table 4**). Correlation analysis between AMF and soil chemical properties showed a positive correlation of sp.*VTX00387* with K content, and a negative correlation with P, AP, and AK contents (**Figure 6**). There was a positive correlation between *S. sinuosa* and the contents of P, AP, and AK, and a negative correlation between *S. sinuosa* and the K content.

### 3.5 Functional prediction of AMF in rhizosphere soil of mango orchard

**Figure 7:**
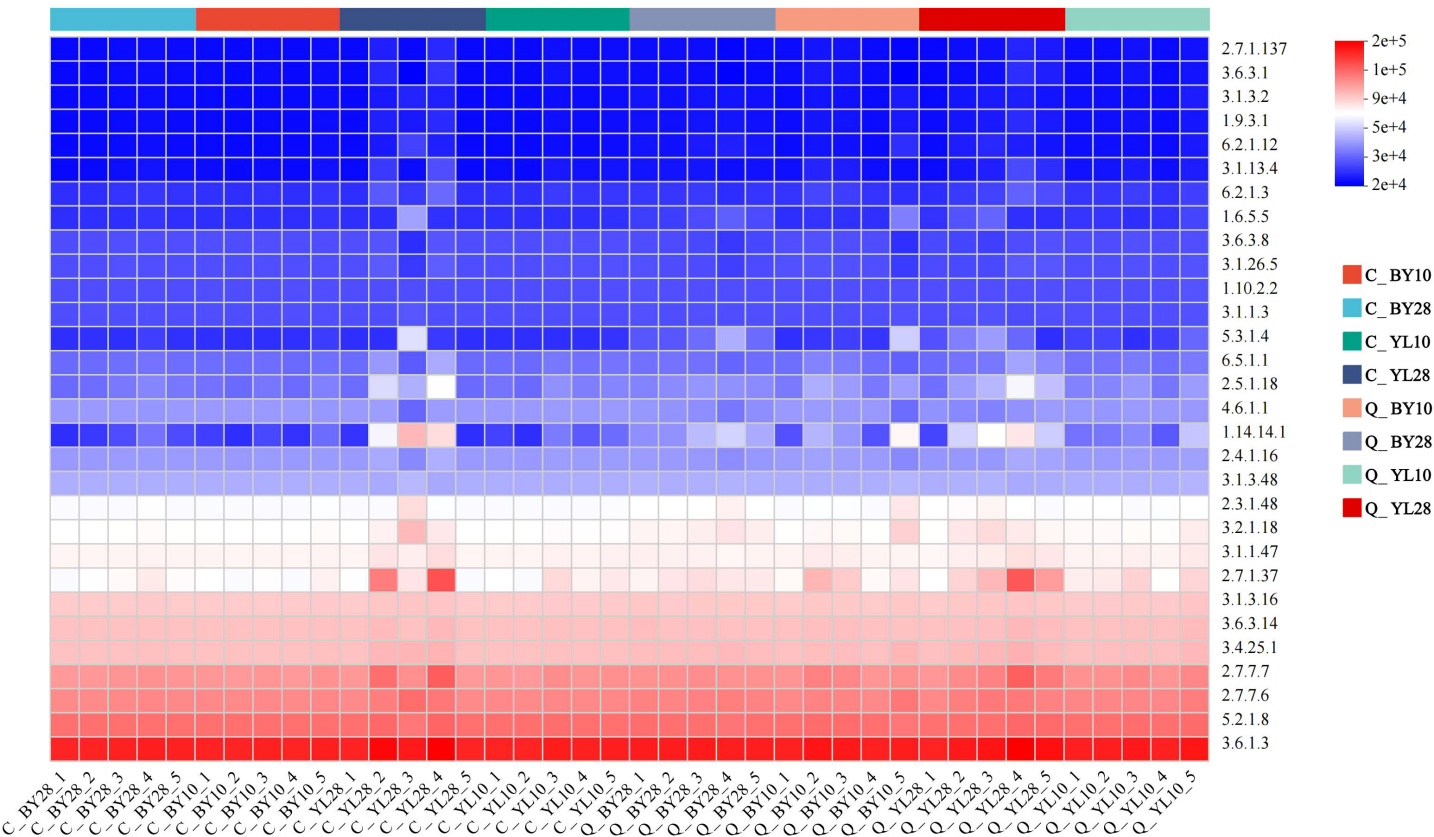
PICRUSt2 functional prediction heatmap of AMF in rhizosphere soil of mango orchard. C_BY10, 10yr old orchard in Baiyu Town during spring; C_BY28, 28yr old orchard in Baiyu Town during spring; C_YL10, 10yr old orchard in Yongle Town during spring; C_YL28, 28yr old orchard in Yongle Town during spring; Q_BY10, 10yr old orchard in Baiyu Town during autumn; Q_BY28, 28yr old orchard in Baiyu Town during autumn; Q_YL10, 10yr old orchard in Yongle Town during autumn; Q_YL28, 28yr old orchard in Yongle Town during autumn.

Based on the 18S rDNA marker gene sequences, PICRUSt2 was used to predict the function of AMF in the rhizosphere soil of mango orchards (**Figure 7**). The results showed that the functions of each AMF sample were mainly enriched by the metabolic pathway 3.6.1.3 (Adenosine Triphosphatase, ATPase). From the perspective of correlation with soil elements, the ATPase function of sp.*VTX00387* was more focused on promoting the absorption, transport, and potassium-related metabolic processes of the K element, thus exhibiting a positive correlation with K content. The ATPase function of *S. sinuosa* was more conducive to the utilization and transformation of phosphorus elements and their related forms (such as AP and AK), and was positively correlated with the contents of P, AP, and AK. This difference in ATPase function towards different soil elements directly leads to their different strategies for resource acquisition.

## 4 Discussion

AMF enhances the absorption of water and nutrients by symbiotic plants through various pathways. In this study we found that N, K, OM and other nutrients content in the 28 year old plantation were significantly higher than those in the 10-year plantation (**Supplementary Table 3**). AMF mycelium can extend into soil pores over time which is difficult for plant roots to reach, thereby establishing a mycorrhizal network and expanding the absorption range of plant roots. The hyphae of AMF have high affinity and can efficiently uptake phosphorus from soil, and transport phosphorus into plant cells through specific transport proteins. In addition, AMF can help plants absorb various nutrients such as N, zinc, and copper, resulting in improved plant growth (Govindan et al., 2020; Zhang et al., 2023). Leguminous plants have a high affinity for AMF and can quickly be infected and establish a good symbiotic relationship with AMF, thereby promoting the formation and function of mycorrhizal networks (Wu and Cui, 2016; Chen and Zeng, 2016). When facing pathogen invasion, microbes can also induce a series of defense responses in plants, activate the expression of defense related genes, synthesize and accumulate disease-related proteins, phytohormones and other substances, and enhance the plant’s disease resistance (Ma et al., 2024; Ye et al., 2024; Hussain et al., 2025).

Currently, the development and utilization of AMF in mangoes is not comprehensive and systematic enough. This study utilized high-throughput amplicon sequencing to decipher AMF community in the rhizosphere soil of two mango orchards from different seasons and different planting years to obtain a comprehensive understanding of AMF community composition. The planting year has a certain impact on the diversity and structure of soil AMF communities. Previous studies showed that the number of AMF OTUs in the rhizospheric soil of three-year-old *Panax notoginseng* was significantly higher than that in the rhizospheric soils of one- and two-year-old *P. notoginseng* (Pei et al., 2023). Similarly, there were significant differences in the richness and community structure of root AMF in *P. notoginseng* at different planting ages. As the planting age increased, the AMF richness was gradually decreased (Yang et al., 2023). We observed the absolute dominance of genus *Glomus* in AMF community (**Figure 3**), which was the dominant genus and the focus of functional AMF development in previously studies (Wang et al., 2011; Song et al., 2020). Chen et al. (2024) also previously found that the dominant AMF genus in the rhizosphere soil of lavender was *Glomus*, and its OTUs number gradually decreased with increasing planting years (Chen et al., 2024). *Glomus* is a common dominant genus in the rhizospheric soil of *Siraitia grosvenorii*, and its average relative abundance increases with the increase in the number of consecutive planting years (Yu et al., 2023). However, we found no significant effect of the planting years on the richness and diversity of AMF in mango rhizosphere soil. This indicates that the AMF community in mango rhizosphere soil can maintain a relatively stable state over a long period of time and did not experience drastic fluctuations due to the extension of planting years. This also implies that AMF has formed a stable adaptive mechanism during its long-term symbiotic evolution with mangoes, which can maintain population size and community structure through its own physiological regulation and ecological strategies.

In this study, *Glomus* had a relative abundance of 53.39% to 86.33% in the mango orchards (**Figure 3**). This may be due to the more diverse reproductive methods of *Glomus*, which gives it a stronger ability to infect plants compared to other genera (Hassan et al., 2011; Govindan et al., 2020; Silvana et al., 2020; Wang et al., 2021), indicating a closer mutualistic relationship between *Glomus* and mangoes compared to other genera. However, the results of dominant AMF species in the rhizosphere soil of mango orchards in different regions were not consistent. The dominant AMF molecular virtual species in the YL sampling point was sp. *VTX00387* belonging to *Glomus* genus, whereas, the dominant species of AMF at the BY sampling point was *S. sinuosa* in the genus *Sclerocystis* (**Figure 4**). These changes in species dominance could be related to different geographical environments or mango orchard management measures (Rasmussen et al., 2018). In addition, many AMF ASVs have not been identified to the species level, and most were belonged to unclassified_f_*Glomeraceae*, indicating that there may be new genera or species belonging to unclassified_f_*Glomeraceae* in the rhizosphere soil of mango orchards.

Geological and climatic conditions are known as the important determining factors affecting AMF communities. However, at the regional scale, soil type, soil pH, and ecological type are the main driving factors. Compared to biological factors (host species), abiotic factors are the most important driving factors determining AMF communities in semi-natural grassland ecosystems in Europe (Van Geel et al., 2017). Vast majority of the AMF studies are mainly performed on annual or perennial herbaceous plants, but there have been relatively less research on the AMF community in the rhizosphere of woody plants, especially fruit trees (mangoes). In this study, there were significant differences in the richness and diversity of AMF in mango rhizosphere soil between sampling points in different regions. Our results found that P, K, AP, and AK had a significant impact on AMF diversity in mango rhizosphere soil (**Figure 6**; **Supplementary Tables 2**, **3**). Among various types of AMF, sp.*VTX00387* showed a positive correlation with soil K content. This means that in areas with abundant soil K content, sp.*VTX00387* can more effectively utilize K elements to meet the needs of its own growth, metabolism, and reproduction, thereby occupying a relatively advantageous position in the microbial community. However, the strain showed a negative correlation with P, AP, and AK content. This phenomenon suggests that sp.*VTX00387* may face growth limitations or intense competitive pressure in soil environments with high P, AP, and AK contents. It is speculated that the reason for this may be that under conditions where these elements are abundant, other microorganisms (such as *S. sinuosa*) have higher utilization efficiency for P, AP, and AK, thereby compressing the living space of sp.*VTX00387* and leading to a decrease in its relative abundance in the corresponding area. On the contrary, *S. sinuosa* is positively correlated with the content of P, AP, and AK, which fully indicates its strong adaptability and competitive advantage in soil environments enriched with these elements, and can fully utilize these resources to maintain its own growth and reproduction. The negative correlation between *S. sinuosa* and K content indicates that it may be at a relative disadvantage in areas with higher K content. This may be due to the relatively weak ability of *S. sinuosa* in K uptake or metabolism, or it may be inhibited by competition from microorganisms with stronger K utilization abilities, such as sp.*VTX00387*. Previously, it was hypothesized that the chemical properties of rhizosphere, as a key ecological factor, have a complex impact on the types, diversity, and richness of AMF in mango rhizosphere soil (IIA, A. E. S, 2021; Zhao et al., 2024). Overall, the structure and composition of AMF community in mango rhizosphere soil were not affected by planting years and seasonal changes, but are mainly influenced by soil chemical properties.

This study found that the distribution pattern of AMF community structure in mango rhizosphere soil (such as dominant AMF molecular virtual species sp.*VTX00387* and *S. sinuosa*) is mainly driven by soil P, K and their available forms (AP, AK), rather than planting years or seasonal changes (**Figure 6**). These results conclusively provide a key ecological basis for the targeted development of AMF in microbial fertilizers. Soil nutrient characteristics should be the core decision-making parameters for microbial agent compatibility and application strategies. From the perspective of future microbial fertilizer development, sp.*VTX00387* can be developed into microbial fertilizers dominated by K elements. For soils with low K content, especially the red soils (pH4.5-5.5) widely distributed in the main mango-producing areas of southern China, the sp.*VTX00387* can be used as the microbial fertilizer component in a soil amendment. To address weak potassium retention capacity caused by the leaching of Ca^2+^ in acidic soils, the sp.*VTX00387* can be applied in conjunction with mineral fertilizer composed of calcium, magnesium and phosphate. Especially in soils with poor potassium mobility, AMF can overcome the diffusion limitations of potassium in the soil through the extension of mycelia, prevent secondary loss of K^+^ resulting from factors like soil solution flow. Such strategy utilizing multi-level synergistic mechanism of activation-absorption-transport can rapidly transports the absorbed K^+^ to the vicinity of the mango root system, making it available for absorption and utilization by the trees. It has the potential to systematically alleviate the supply-demand contradiction of K elements in the acidic soils of the mango-producing areas in southern China. Similarly, *S. sinuosa* can be developed to a microbial fertilizer with P as the main element, while balancing K. In some soils with high P contents but low utilization efficiency, such as those where long-term application of P fertilizer leads to its accumulation, the use of microbial fertilizers containing *S. sinuosa* can promote the transformation and utilization of insoluble P in the soil, improve the utilization efficiency of soil P resources, reduce P waste and environmental pollution (Cruz-Paredes et al., 2021). This addresses the issue of high phosphorus content and low utilization in acidic soil by relying on mycelial networks to achieve the synergy of phosphorus and potassium, providing microbial solutions for the sustainable nutrient management in southern China mango plantations. Although sp.*VTX00387* and *S. sinuosa* have shown clear correlation with soil P and K, their future commercialization still requires the establishment of a life cycle assessment system (LCA) for AMF functional strains, the development of soil nutrient AMF community, crop phenotype intelligent matching algorithms, and the precise release of microbial agents.

## 5 Conclusion

Our results showed that planting years and seasonal changes had minor impact on the structure and composition of AMF in mango rhizosphere soil, while soil chemical properties such as P, K, and their available content were major factors correlating with the AMF composition at two different mango orchards. A total of 733 ASVs were obtained from the rhizosphere soil of mango orchards, and 7 species of AMF belonging to 5 genera and 3 families were identified. Among them, *Glomus* was the absolute dominant genus in the mango AMF community, with a relative abundance of 53.39%~86.33%, followed by *Sclerocystis*. Differentiation analysis indicated that the molecular virtual species sp.*VTX00387* in the *Glomus* genus and *S. sinuosa* in the *Sclerocystis* genus were the dominant AMF species in the mango rhizosphere. The sp.*VTX00387* was positively correlated with K content and negatively correlated with P, AP, and AK. The high abundance of sp.*VTX00387* and *S. sinuosa* in the rhizosphere make them ideal candidate for specialized microbial fertilizers. This study provides a theoretical basis and a reserve of strain resources for regulating nutrient dynamics in mango orchards to improve mango trees health and productivity.

## Data Availability

The datasets presented in this study can be found in online repositories. The names of the repository/repositories and accession number(s) can be found in the article/**Supplementary Material**.
