# Supplementary material for: Structure and composition of arbuscular mycorrhizal fungal community associated with mango
Source: Front Plant Sci. 2025 May 8;16:1578936. doi: 10.3389/fpls.2025.1578936 (PMC12095367; doi:10.3389/fpls.2025.1578936)
Supplement: Supplementary file 2 [file DataSheet2.docx]

**Structure and composition of arbuscular mycorrhizal fungal community associated with mango**

CuifengYang^1,2,3,#^, ZhengTeng^1,2,3,#^, ZhiboJin^1,2,3^, QiufeiOuyang^1,2,3^, Lingling Lv^1,2,3^, XianbinHou^1,2,3^, Muzammil Hussain^1,2,3^, ZhengjieZhu^1,2,3,^*

^1^College of Agriculture and Food Engineering, Baise University, Baise 533000, China

^2^Guangxi Key Laboratory of Biology for Mango, Baise 533000, China

^3^College of Subtropical Characteristic Agricultural Industry, Baise 533000, China

^#^These authors contributed equally to this work.

**Corresponding author Email:** [zhuzhjie@163.com](mailto:zhuzhjie@163.com) (Zhengjie Zhu)

**Supplementary Table: 4**

**Supplementary Figures: 5**

Supplementary Table 1. Name Information of Mango Rhizosphere Soil Samples

| Name | Sample Name | Name | Sample Name | Name | Sample Name | Name | Sample Name |
| --- | --- | --- | --- | --- | --- | --- | --- |
| 1 | C_BY28_1 | 11 | C_YL28_1 | 21 | Q_BY28_1 | 31 | Q_YL28_1 |
| 2 | C_BY28_2 | 12 | C_YL28_2 | 22 | Q_BY28_2 | 32 | Q_YL28_2 |
| 3 | C_BY28_3 | 13 | C_YL28_3 | 23 | Q_BY28_3 | 33 | Q_YL28_3 |
| 4 | C_BY28_4 | 14 | C_YL28_4 | 24 | Q_BY28_4 | 34 | Q_YL28_4 |
| 5 | C_BY28_5 | 15 | C_YL28_5 | 25 | Q_BY28_5 | 35 | Q_YL28_5 |
| 6 | C_BY10_1 | 16 | C_YL10_1 | 26 | Q_BY10_1 | 36 | Q_YL10_1 |
| 7 | C_BY10_2 | 17 | C_YL10_2 | 27 | Q_BY10_2 | 37 | Q_YL10_2 |
| 8 | C_BY10_3 | 18 | C_YL10_3 | 28 | Q_BY10_3 | 38 | Q_YL10_3 |
| 9 | C_BY10_4 | 19 | C_YL10_4 | 29 | Q_BY10_4 | 39 | Q_YL10_4 |
| 10 | C_BY10_5 | 20 | C_YL10_5 | 30 | Q_BY10_5 | 40 | Q_YL10_5 |

Note: "C" represents spring, "Q" represents autumn, "BY" represents mango orchard in Baiyu Town, "YL" represents mango orchard in Yongle Town, "10" represents mango planting years of 10 years, and "28" represents mango planting years of 28 years.

Supplementary Table 2. Raw data on chemical properties of rhizosphere soil in mango orchard

| SampleID | pH | OM（g/kg） | N  （g/kg） | P  （g/kg） | K  （g/kg） | HN（mg/kg） | AP（mg/kg） | AK（mg/kg） |
| --- | --- | --- | --- | --- | --- | --- | --- | --- |
| C_BY28_1 | 5.68 | 31.93 | 1.76 | 1.03 | 9.30 | 141 | 59.6 | 228 |
| C_BY28_2 | 4.89 | 13.76 | 0.76 | 0.30 | 8.53 | 49 | 4.0 | 204 |
| C_BY28_3 | 5.39 | 22.02 | 1.27 | 0.37 | 8.83 | 93 | 2.3 | 74 |
| C_BY28_4 | 5.29 | 25.88 | 1.54 | 0.56 | 8.99 | 120 | 15.7 | 145 |
| C_BY28_5 | 5.03 | 23.11 | 1.29 | 0.38 | 8.78 | 98 | 3.2 | 144 |
| C_BY10_1 | 4.92 | 19.27 | 1.11 | 0.66 | 9.45 | 80 | 38.7 | 250 |
| C_BY10_2 | 5.22 | 19.26 | 1.08 | 0.63 | 7.50 | 80 | 41.8 | 237 |
| C_BY10_3 | 4.89 | 15.42 | 0.92 | 0.48 | 7.76 | 68 | 23.6 | 234 |
| C_BY10_4 | 4.72 | 14.87 | 0.96 | 0.62 | 8.98 | 73 | 23.7 | 193 |
| C_BY10_5 | 4.75 | 15.41 | 0.94 | 0.53 | 8.94 | 67 | 16.6 | 177 |
| C_YL28_1 | 4.88 | 24.76 | 1.62 | 0.35 | 13.84 | 110 | 2.9 | 100 |
| C_YL28_2 | 5.10 | 20.36 | 1.29 | 0.64 | 12.65 | 96 | 51.0 | 84 |
| C_YL28_3 | 4.27 | 18.73 | 1.45 | 0.57 | 13.92 | 175 | 45.8 | 508 |
| C_YL28_4 | 5.26 | 23.13 | 1.55 | 1.18 | 13.80 | 105 | 90.6 | 124 |
| C_YL28_5 | 4.81 | 17.06 | 1.17 | 0.34 | 12.44 | 79 | 5.3 | 82 |
| C_YL10_1 | 4.90 | 19.81 | 1.40 | 0.27 | 14.65 | 91 | 0.9 | 56 |
| C_YL10_2 | 4.71 | 20.92 | 1.21 | 0.23 | 9.57 | 86 | 1.5 | 40 |
| C_YL10_3 | 4.83 | 18.16 | 1.07 | 0.23 | 8.86 | 86 | 1.7 | 47 |
| C_YL10_4 | 4.98 | 21.99 | 1.39 | 0.42 | 12.04 | 100 | 4.9 | 51 |
| C_YL10_5 | 4.88 | 24.21 | 1.60 | 0.30 | 12.68 | 118 | 2.0 | 96 |
| Q_BY28_1 | 4.9 | 16.5 | 1.01 | 0.54 | 7.5 | 77 | 32.5 | 161 |
| Q_BY28_2 | 5.0 | 17.6 | 1.22 | 0.80 | 9.0 | 98 | 63.5 | 282 |
| Q_BY28_3 | 5.5 | 14.5 | 1.06 | 0.74 | 9.4 | 77 | 37.0 | 127 |
| Q_BY28_4 | 5.1 | 20.6 | 1.44 | 1.14 | 8.6 | 123 | 120.6 | 182 |
| Q_BY28_5 | 4.7 | 11.4 | 0.85 | 0.52 | 9.0 | 55 | 19.0 | 185 |
| Q_BY10_1 | 5.6 | 16.4 | 1.18 | 0.77 | 7.2 | 94 | 32.4 | 195 |
| Q_BY10_2 | 5.4 | 13.1 | 1.03 | 0.93 | 8.9 | 76 | 61.3 | 155 |
| Q_BY10_3 | 5.0 | 11.1 | 0.90 | 0.66 | 8.7 | 63 | 32.7 | 123 |
| Q_BY10_4 | 5.3 | 12.5 | 0.98 | 1.56 | 8.9 | 73 | 53.0 | 248 |
| Q_BY10_5 | 5.2 | 16.4 | 1.17 | 0.86 | 8.7 | 84 | 47.7 | 233 |
| Q_YL28_1 | 4.8 | 20.9 | 1.60 | 0.46 | 12.8 | 127 | 36.2 | 135 |
| Q_YL28_2 | 4.8 | 17.0 | 1.19 | 0.28 | 10.2 | 91 | 4.3 | 63 |
| Q_YL28_3 | 5.4 | 26.5 | 1.83 | 0.51 | 12.4 | 145 | 8.7 | 81 |
| Q_YL28_4 | 5.4 | 16.4 | 1.29 | 0.53 | 13.9 | 82 | 30.1 | 106 |
| Q_YL28_5 | 4.9 | 15.3 | 1.33 | 0.50 | 13.0 | 100 | 21.1 | 158 |
| Q_YL10_1 | 4.9 | 17.0 | 1.27 | 0.32 | 11.3 | 83 | 1.5 | 48 |
| Q_YL10_2 | 5.1 | 17.6 | 1.25 | 0.36 | 8.5 | 94 | 4.4 | 47 |
| Q_YL10_3 | 5.0 | 13.1 | 1.04 | 0.28 | 9.7 | 75 | 1.7 | 32 |
| Q_YL10_4 | 5.0 | 16.5 | 1.23 | 0.27 | 11.2 | 84 | 1.5 | 33 |
| Q_YL10_5 | 5.0 | 14.2 | 1.29 | 0.37 | 12.6 | 79 | 2.9 | 61 |

Supplementary Table 3. Chemical properties of rhizosphere soil in mango orchard

| soil chemical property | pH | OM（g/kg） | N（g/kg） | P（g/kg） |
| --- | --- | --- | --- | --- |
| C_BY28 | 5.26±0.14a | 23.34±2.95a | 1.32±0.17abcd | 0.53±0.13bcd |
| C_BY10 | 4.90±0.09b | 16.85±0.99bc | 1.00±0.04e | 0.58±0.03bc |
| C_YL28 | 4.86±0.17b | 20.81±1.41ab | 1.42±0.08a | 0.62±0.15b |
| C_YL10 | 4.86±0.04b | 21.02±1.02ab | 1.33±0.09abc | 0.29±0.04d |
| Q_BY28 | 5.04±0.13ab | 16.12±1.54cd | 1.12±0.10cde | 0.75±0.11ab |
| Q_BY10 | 5.30±0.10a | 13.90±1.07d | 1.05±0.05de | 0.96±0.16a |
| Q_YL28 | 5.06±0.14ab | 19.22±2.05abc | 1.45±0.12a | 0.46±0.05bcd |
| Q_YL10 | 5.00±0.03ab | 15.68±0.86cd | 1.22±0.05abcde | 0.32±0.02cd |

Continuing from the previous table

| soil chemical property | K（g/kg） | HN（mg/kg） | AP（mg/kg） | AK（mg/kg） |
| --- | --- | --- | --- | --- |
| C_BY28 | 8.89±0.13d | 100.20±15.39abc | 16.96±10.94bc | 159.00±26.86ab |
| C_BY10 | 8.53±0.38d | 73.60±2.80c | 28.88±4.84abc | 218.20±14.05a |
| C_YL28 | 13.33±0.32a | 113.00±16.37a | 39.12±16.27ab | 179.60±82.44ab |
| C_YL10 | 11.56±1.06bc | 96.20±6.02abc | 2.20±0.70c | 58.00±9.85c |
| Q_BY28 | 8.70±0.33d | 86.00±11.48abc | 54.52±18.03a | 187.40±25.82ab |
| Q_BY10 | 8.48±0.32d | 78.00±5.22c | 45.42±5.68ab | 190.80±23.40ab |
| Q_YL28 | 12.46±0.62ab | 109.00±11.73ab | 20.08±6.08bc | 108.60±17.30bc |
| Q_YL10 | 10.66±0.71c | 83.00±3.18bc | 2.40±0.56c | 44.20±5.38c |

Note: Lowercase English letters indicate significant differences (p<0.05). C_BY10, 10yr old orchard in Baiyu Town during spring; C_BY28, 28yr old orchard in Baiyu Town during spring; C_YL10, 10yr old orchard in Yongle Town during spring; C_YL28, 28yr old orchard in Yongle Town during spring; Q_BY10, 10yr old orchard in Baiyu Town during autumn; Q_BY28, 28yr old orchard in Baiyu Town during autumn; Q_YL10, 10yr old orchard in Yongle Town during autumn; Q_YL28, 28yr old orchard in Yongle Town during autumn.

Supplementary Table 4. db-RDA table of AMF and soil chemical properties in the rhizosphere of mango orchard

| soil chemical property | CAP1 | CAP2 | r^2^ | P_value |
| --- | --- | --- | --- | --- |
| pH | -0.5195 | -0.8545 | 0.1172 | 0.107 |
| OM | 0.9893 | -0.1457 | 0.0963 | 0.157 |
| N | 0.993 | -0.1177 | 0.2814 | 0.003 |
| P | -0.9549 | 0.2969 | 0.0666 | 0.286 |
| K | 0.9976 | -0.0687 | 0.5418 | 0.001 |
| HN | 0.9915 | -0.1303 | 0.1742 | 0.039 |
| AP | -0.8683 | 0.496 | 0.0615 | 0.325 |
| AK | -0.912 | -0.4103 | 0.1048 | 0.132 |

**
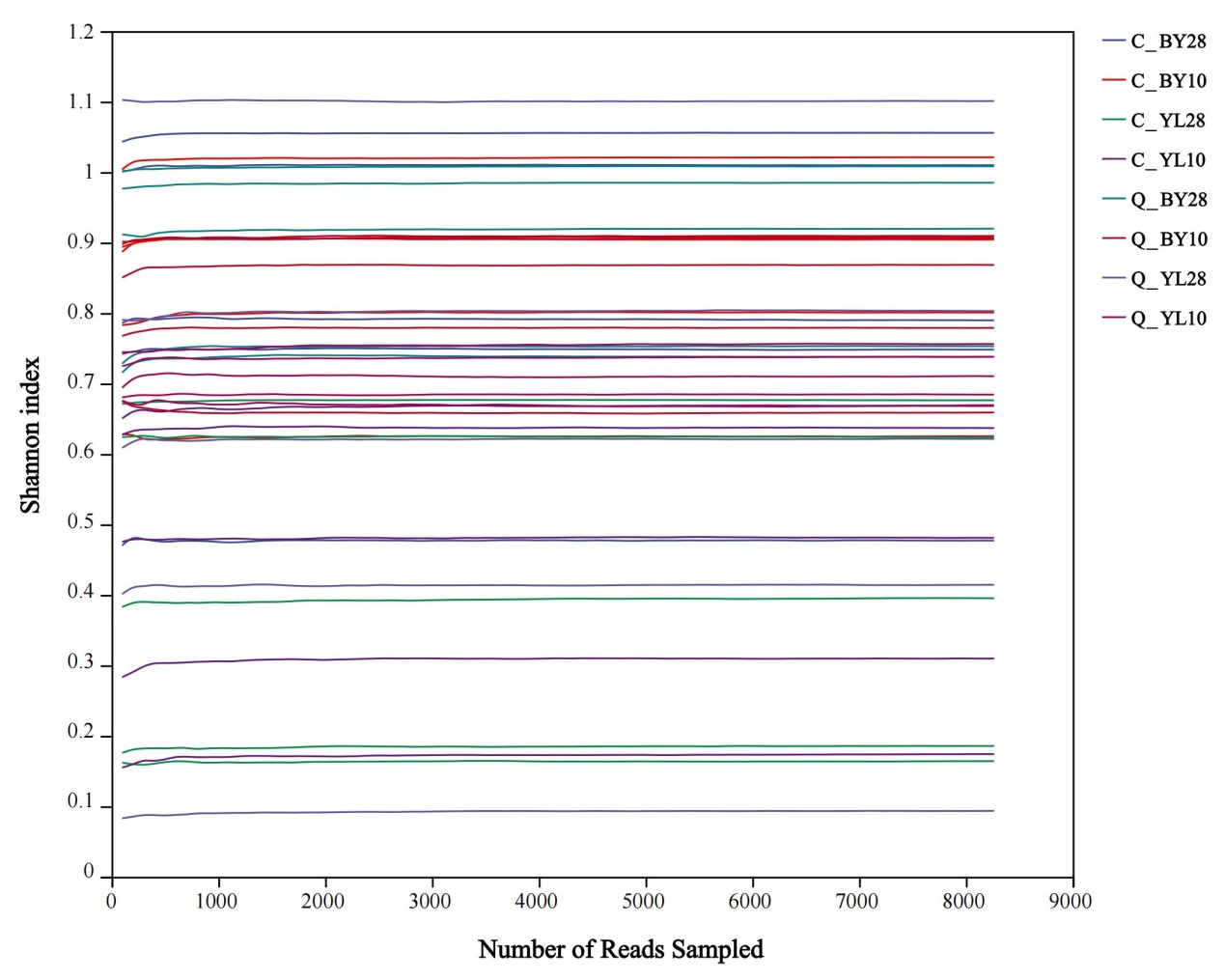
**

**Figure S1.** Rarefaction curves analysis of AMF in rhizosphere soil of mango orchard.

**
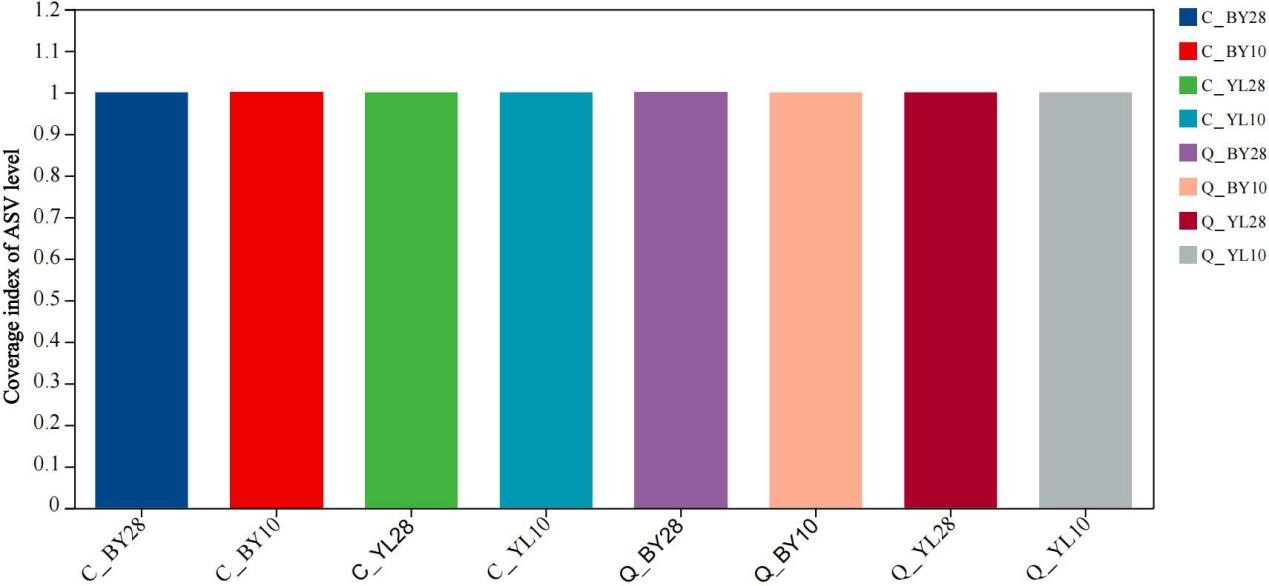
**

**Figure S2.** Coverage index of AMF in rhizosphere soil of mango orchard.


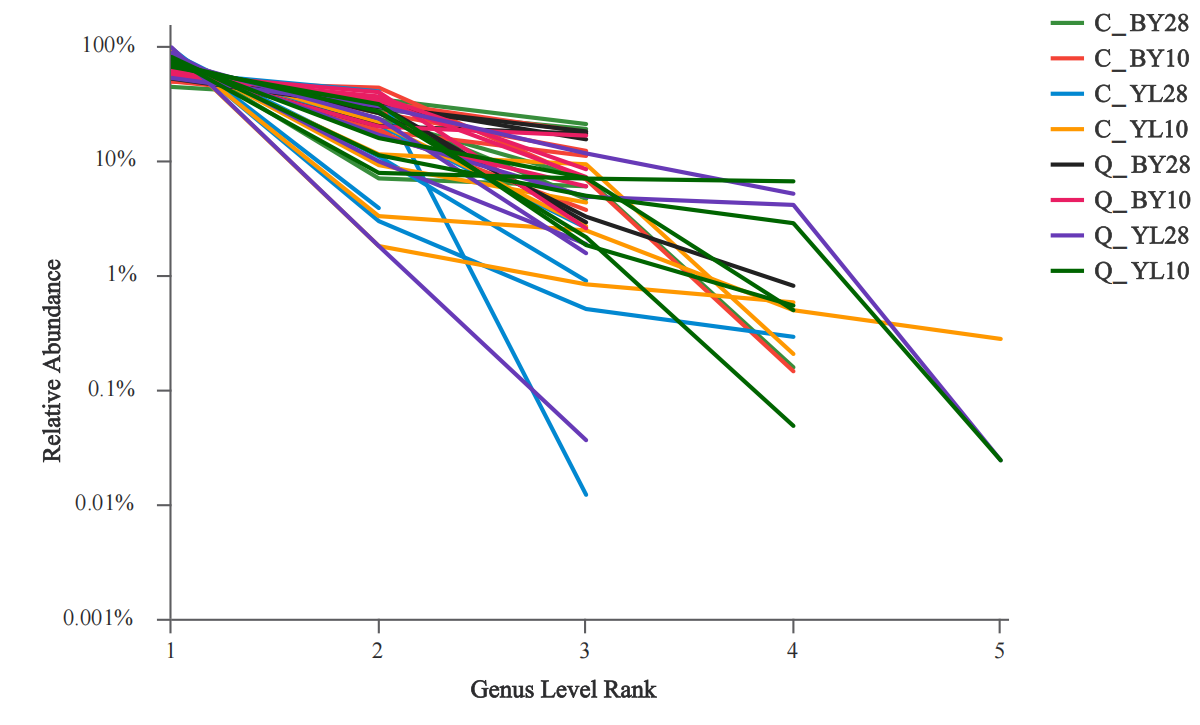


**Figure S3.** Rank abundance curve of AMF in rhizosphere soil of mango orchard.

**A**

**
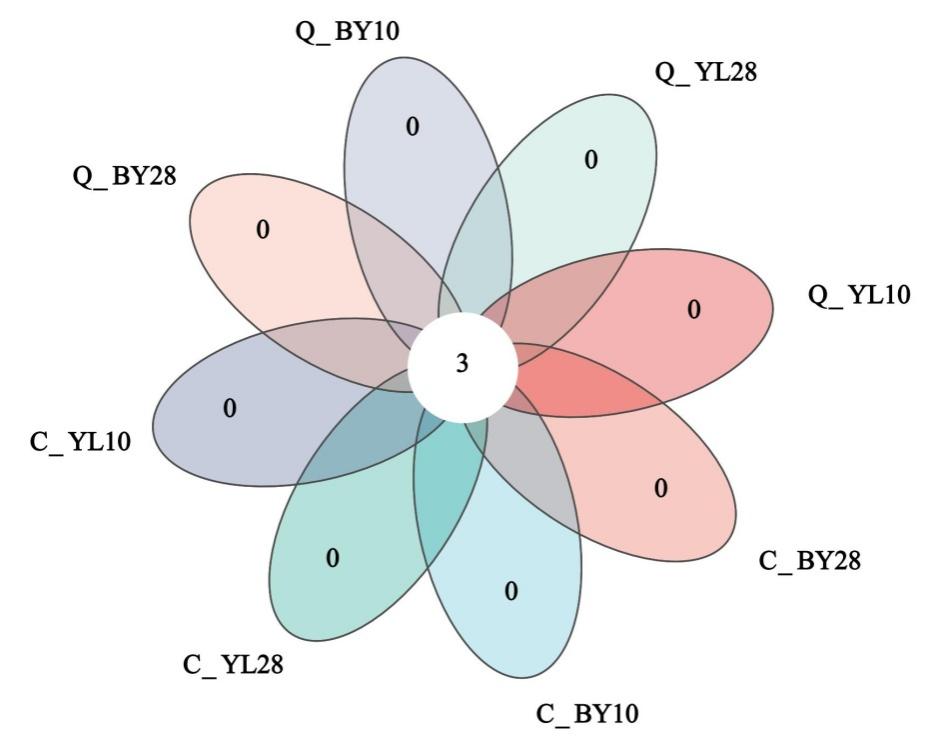
**

**B**

**
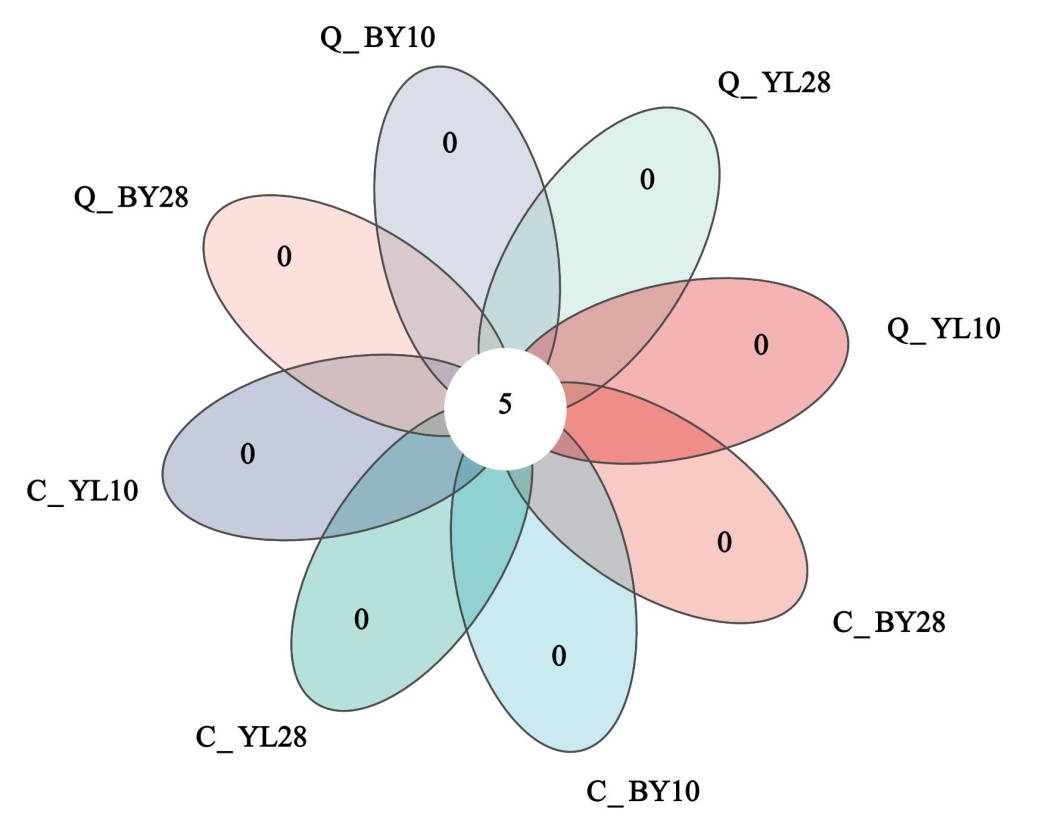
**

**Figure S4.Venn diagram of AMF microbial community in rhizosphere soil of mango orchard at the (A) genus level and (B) species level.**

**A**

**
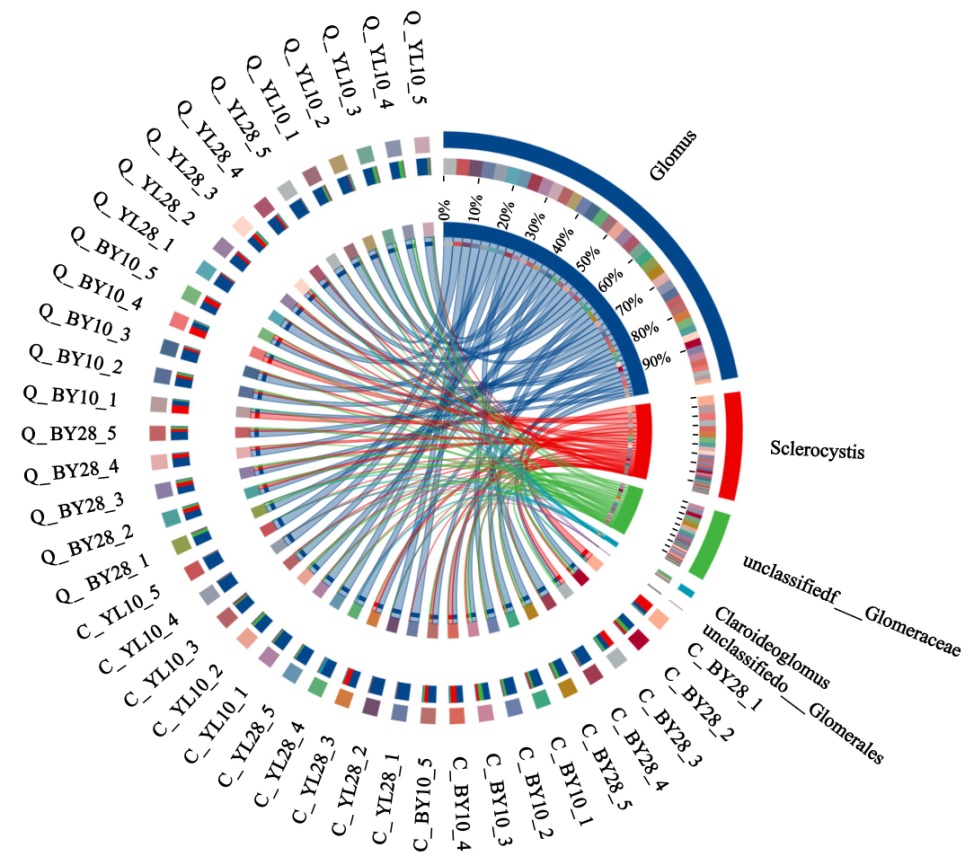
**

**B**

**
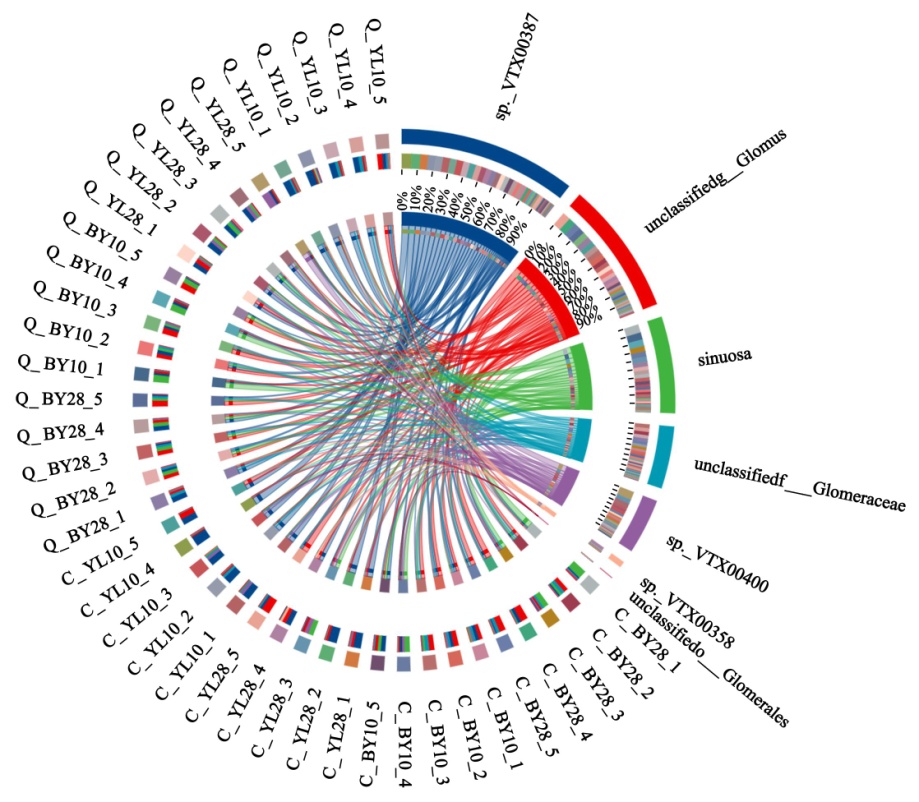
**

**Figure S5. Circos diagram of AMF microbial community in rhizosphere soil of mango orchard at the (A) genus level and (B) species level.**
